# Illuminating Genetic Diversity and Selection Signatures in Matou Goats through Whole-Genome Sequencing Analysis

**DOI:** 10.3390/genes15070909

**Published:** 2024-07-12

**Authors:** Ruiyao HuangFu, Haobang Li, Yang Luo, Fang He, Cheng Huan, Zulfiqar Ahmed, Baizhong Zhang, Chuzhao Lei, Kangle Yi

**Affiliations:** 1 Hunan Institute of Animal and Veterinary Science, Changsha 410131, China; lhb.m2002@163.com (H.L.); xinhelu509@163.com (Y.L.); hf3893@126.com (F.H.); 15173116399@163.com (C.H.); zhangbz6181@163.com (B.Z.); 2 Key Laboratory of Animal Genetics, Breeding and Reproduction of Shaanxi Province, College of Animal Science and Technology, Northwest A&F University, Yangling 712000, China; leichuzhao1118@126.com; 3 Faculty of Veterinary and Animal Sciences, University of Poonch Rawalakot, Rawalakot 12350, Pakistan; zulfiqarahmed@upr.edu.pk

**Keywords:** genetic diversity, Matou goat, selection signature, whole genome

## Abstract

(1) Background: Matou goats, native to Hunan and Hubei provinces in China, are renowned for their exceptional meat and skin quality. However, a comprehensive whole-genome-based exploration of the genetic architecture of this breed is scant in the literature. (2) Methods: To address this substantial gap, we used whole-genome sequences of 20 Matou goats and compared them with published genomic data of 133 goats of different breeds across China. This comprehensive investigation sought to assess genetic diversity, population structure, and the presence of genomic selection signals. (3) Results: The whole genome of Matou goat populations yielded a substantial catalog of over 19 million single nucleotide polymorphisms (SNPs), primarily distributed within intergenic and intron regions. The phylogenetic tree analysis revealed distinct clades corresponding to each goat population within the dataset. Notably, this analysis positioned Matou goats in a closer genetic affinity with Guizhou White goats, compared to other recognized goat breeds. This observation was corroborated by principal component analysis (PCA) and admixture analysis. Remarkably, Matou goats exhibited diminished genetic diversity and a notable degree of inbreeding, signifying a reduced effective population size. Moreover, the study employed five selective sweep detection methods (including PI, CLR, PI-Ratio, Fst, and XP-EHH) to screen top signal genes associated with critical biological functions, encompassing cardiomyocytes, immunity, coat color, and meat quality. (4) Conclusions: In conclusion, this study significantly advances our understanding of the current genetic landscape and evolutionary dynamics of Matou goats. These findings underscore the importance of concerted efforts in resource conservation and genetic enhancement for this invaluable breed.

## 1. Introduction

Goats, one of the earliest domesticated mammals, played a vital role in ancient societies by providing meat, milk, wool, and fur, fostering societal progress [1]. Archaeological findings suggest that the domestication of goats from the Bezoar (*Capra aegagrus*) occurred approximately 10,000 years ago in the Fertile Crescent of the Near East [2]. Genome-wide analyses have categorized domestic goats into four continental groups: EUR (European), AFR (African), SWA-SAS (Southwest Asian), and EAS (East Asian) [3]. Numerous studies, utilizing whole-genome sequencing, have explored economically relevant traits in goat genetics, such as litter size, heat, and disease resistance [4,5]. As a result, the investigation and exploitation of these genes offer a solid scientific foundation for enhancing goat adaptability and productivity in genetic breeding [6,7].

China, being a major hub for goat husbandry, holds the top position globally in terms of goat stock, slaughtering rate, and meat production. Within China, the Matou goat, predominantly found in Hunan and Hubei provinces, stands out as a distinguished local breed renowned for its exceptional attributes in meat and skin quality. These goats exhibit high slaughter rates, prolific reproduction, early sexual maturity, rapid growth, substantial body size, robust disease resistance, resilience to roughage-based diets, ease of maintenance, and various other commendable production traits [8]. Notably, the Heifer International Foundation designated the Matou goat as the preferred meat breed in Asia in 1992 [9].

In previous studies, efforts were made to evaluate the genetic diversity and relationships of seven Chinese indigenous meat goat breeds and an African goat breed, as well as to determine the genetic relationships among five Chinese indigenous goat breeds [10,11]. However, these studies primarily relied on standard microsatellite markers (STRs). There is currently limited information available on the whole-genome analysis of the Matou goat. Single nucleotide polymorphisms (SNPs) are the most common heritable variants in humans and they are highly stable in the genome, accounting for over 90% of all known polymorphisms. When compared to STRs, SNPs offer a greater density, increased representativeness, and strong genetic stability across the entire genome. Whole-genome sequencing enables the acquisition of high-throughput and accurate DNA sequences of the entire genome. In recent years, the rapid advancements in high-throughput sequencing technology have made whole-genome resequencing an essential tool for investigating the origin, domestication, migration, historical dynamics, and key trait-related functional genes in livestock and poultry [12]. Therefore, employing the whole genome for studying the genetic diversity of the Matou goat holds significant importance.

Despite its historical significance, the Matou goat has faced challenges stemming from inadequate management and planning, resulting in a substantial decline in population numbers and overall breed quality in many of its inhabiting regions. While recent years have witnessed increased efforts from both state and local governments to protect and rejuvenate the Matou goat breed, comprehensive genomic studies on its germplasm characteristics and genetic features have remained conspicuously limited [13,14].

This study was conducted to address this research gap by conducting whole-genome resequencing of 20 Matou goats, utilizing the *Capra hircus* reference genome as a reference. Additionally, data from 133 previously published goat genomes were incorporated to investigate genetic variations, delineate population structures, and perform selective signal scanning analyses. The outcomes of this research are anticipated to yield valuable insights into the current status of genomic diversity and selective characteristics of the Matou goat, serving as a foundation for resource conservation and genetic enhancement initiatives for this unique breed.

## 2. Materials and Methods

### 2.1. Ethics Approval Statement

This study obtained ethical approval from the Institutional Animal Care and Use Committee at Northwest A&F University (permit number: NWAFAC1019), in strict adherence to China’s regulatory framework for experimental animals. By upholding the welfare and safety of the animals involved, specific consent procedures were not deemed necessary, as we rigorously followed the National Standard for the Care and Use of Laboratory Animals.

### 2.2. Sampling and Illumina Whole-Genome Sequencing

Ear tissue samples from a group of Matou goats (n = 20) were collected from native breeding tracts (Shimen County, Changde City, Hunan Province, China) for genomic investigation. These goats were all kept in the same group to ensure a consistent breeding environment, and collected from male animals.

Genomic DNA extraction was carried out using a conventional phenol/chloroform-based protocol previously described by [15]. (This method follows the steps outlined in DNA extraction, utilizing special kits purchased by the laboratory. In this specific case, the kit used is from TIANGEN BIOTECH (BEIJING) Co., Ltd. Beijing, China) The quality and integrity of the DNA were ensured through Nandrop spectrophotometer and gel electrophoresis. Individual DNA libraries were constructed for each sample, each characterized by a 500 bp insert size. Subsequently, high-throughput sequencing was performed employing the Illumina NovaSeq 6000 platform, employing a 2 × 150 bp sequencing model, at the Novogene Bioinformatics Institute, Beijing, China. This approach yielded 150-bp paired-end sequence data of high precision and coverage.

**Figure 1:**
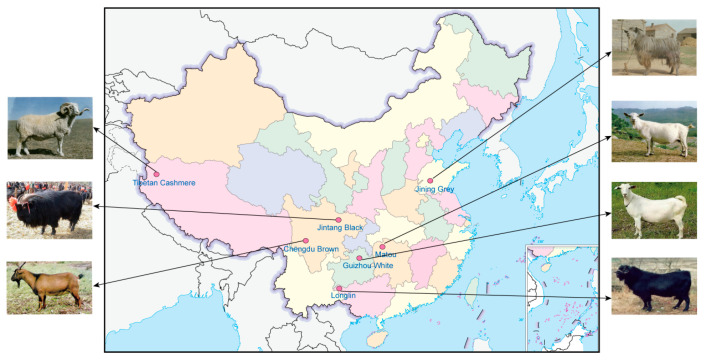
Geographical distribution of seven species of goats in China included in the dataset.

In addition to the Matou goat dataset, we augmented our analysis with genomic data from 133 goats of various breeds, encompassing Chengdu Brown Goats (n = 15), Longlin Goats (n = 20), Jining Grey Goats (n = 34), Guizhou White Goats (n = 5), Tibetan Cashmere Goats (n = 14), and Jintang Black Goats (n = 45). Collectively, our study encompassed a dataset comprising 153 goats, enhancing the comprehensiveness and scope of our genetic investigation. An additional 133 published genomic data used in this study were downloaded from NCBI. The raw FASTQ sequences for the Matou goats have been deposited to the NCBI under the BioProject accession number PRJNA1046221. The detailed sample information including geographic distribution is presented in (Figure 1 and Table S2).

### 2.3. Alignment and SNP Calling

In our genomic analysis pipeline, we employed Trimommatic v.0.39 to perform initial quality-based trimming of raw reads. Subsequently, the resulting clean reads were aligned to the goat reference assembly (ARS1.2) using the default parameters of BWA-MEM (v.0.7.13-r1126) [16]. Further refinement included sorting the Bam files with SAMtools and employing ‘MarkDuplicates’ from Picard v.2.20.2 to identify potential PCR duplicates within the mapped reads, followed by their subsequent organization using Picard software. To facilitate the identification of single nucleotide polymorphisms (SNPs), we generated GVCF (Genomic Variant Call Format) files utilizing ‘HaplotypeCaller’ in GATK, specifically employing the ‘ERC GVCF’ option. Subsequently, we employed ‘GenotypeGVCFs’ and ‘SelectVariants’ to extract potential SNPs from the amalgamated GVCF files. Stringent quality control measures were applied to ensure precision, including GATK’s Variant Filtration with parameters such as ‘-cluster Size 3’ and ‘-cluster Window 10’ to mitigate false positive calls. Additionally, our SNP filtering criteria encompassed specific thresholds, such as mean depth ≥ 3, quality by depth (QD) ≥ 2, strand odds ratio (SOR) > 3, Fisher strand (FS) > 60, mapping quality (MQ) ≥ 40, mapping quality rank sum test (MQRankSum) ≥ 12.5, and read position rank sum test (ReadPosRankSum) ≥ 8. Non-biallelic SNPs and those with a genotype missing rate exceeding 10% were meticulously excluded from the analysis. The resultant set of high-quality SNPs was subsequently annotated based on their genomic positions, utilizing Annovar v4.7 [17] in conjunction with the reference goat assembly, enriching our dataset with comprehensive genomic insights.

### 2.4. Population Structure and Phylogenetic Analysis

To elucidate the population structure and evolutionary relationships among our dataset, we conducted a series of analytical procedures. First, a set of SNPs was derived following data pruning in PLINK v1.9, utilizing the parameters (-indep-pair-wise 50 5 0.2). Subsequently, we constructed an unrooted neighbor-joining (NJ) tree based on the matrix of pairwise genetic distances. This tree was generated employing MEGA v7.0 [18] and visualized using iTOL (https://itol.embl.de/ accessed on 16 October 2023). Additionally, a principal component analysis (PCA) was carried out using the smartPCA function from the EIGENSOFT v5.0 package [19] to provide further insights into the population structure. To comprehensively assess the population structure, we utilized ADMIXTURE v1.3 [20] to estimate genetic clusters (K) ranging from 2 to 7. These analyses collectively enhance our understanding of the genetic relationships and structure within our study population.

### 2.5. Genetic Diversity and Linkage Disequilibrium Detection

To assess nucleotide diversity within each breed, we employed VCFtools v0.1.16, utilizing window sizes of 50 kb with a 20 kb step size increment [21]. Furthermore, we quantified and visualized linkage disequilibrium (LD) decay concerning the physical distance between single nucleotide polymorphisms (SNPs) using PopLDdecay v3.42 software, utilizing default parameters [22].

To investigate the presence of runs of homozygosity (ROH) within each goat breed, we utilized PLINK v1.9 [23] with the following command settings: ‘--chr-set 29 --homozyg-window-snp 200 --homozyg-snp 200 --homozyg-kb 100 --homozyg-gap 1000 --homozyg-window-threshold 0.05 --homozyg-window-het 1’. Subsequently, we generated scatter plots illustrating both the number and length of ROH for each breed, utilizing R v4.3.1.

### 2.6. Selective Sweep Identification

To identify selection signatures driven by artificial selection and genetic adaptation, we conducted genome scans within and between Matou goat populations and Jining Grey goat populations. Within Matou goats, we used the following two statistical tools: nucleotide diversity (θπ) and composite likelihood ratio (CLR) [24]. θπ was calculated in 50 kb windows with a 20 kb step using VCFtools v0.1.16 [25]. CLR scores, computed in non-overlapping 50 kb windows, were obtained using SweepFinder2 v1.0 [26]. Empirical *p*-values were computed for both methods, and the top 1% of overlapping windows were considered candidate selection signatures.

Comparing Matou and Jining Grey goats, we employed fixation index (Fst), genetic diversity (π-ratio), and cross-population extended haplotype homozygosity (XP-EHH). Fst and π-ratio were analyzed using VCFtools with a 50 kb window and a 20 kb step. XP-EHH statistics were computed with SELSCAN V1.1 [25,26], and significant genomic regions were defined by a *p*-value < 0.01 threshold. Genomic regions identified by at least two of the three methods (Fst, π-ratio, XP-EHH) were considered candidate regions of positive selection [27]. To further rigor the analysis of selection candidate genes functions we computed Tajima’s D statistic with VCFtools candidate genes [28].

### 2.7. Candidate Gene Enrichment Analysis

To explore the candidate genes’ functions and signaling pathways, we also performed GO and KEGG pathway enrichment analysis through an online tool available at https://david.ncifcrf.gov/tools.jsp (accessed on 30 October 2023).

## 3. Results

### 3.1. Sequence Read Depths and Variants Landscape

Alignment of clean reads of whole-genome samples of Matou goats with C. Hircus reference genome (ARS 1.2) yielded 4.81 billion (4.81 × 10^9^) clean reads with sequence coverage depths of 13.06 × and 19.99 million (19.99 × 10^6^) of total biallelic SNPs (Tables S1 and S3). Most of the detected SNPs were found in the intergenic and intron regions (60.04% and 36.03%, respectively). Exons accounted for 1.65%, including 57,281 nonsynonymous SNPs and 78,386 synonymous SNPs).

### 3.2. Population Structure Analysis

**Figure 2:**
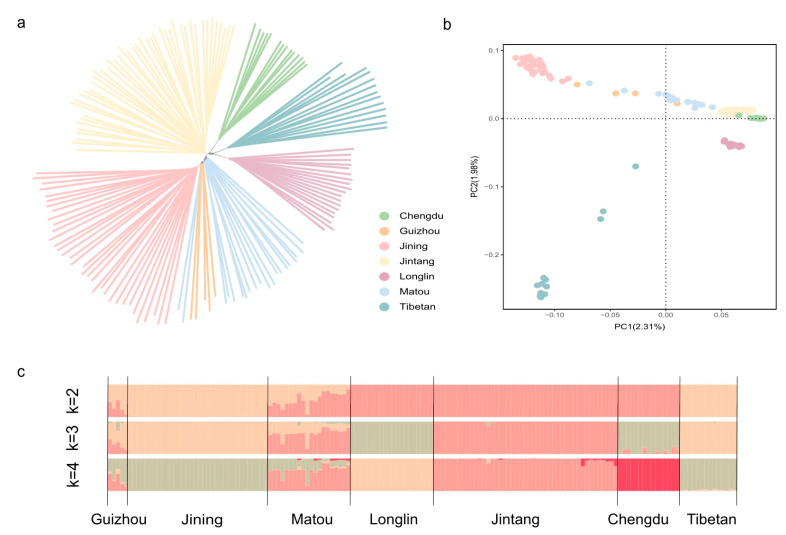
Population genetic analysis of Matou goat: (**a**) neighbor-joining tree of relationships among seven populations; (**b**) principal component analysis of goat with PC1 against PC2; (**c**) genetic structure of goat using ADMIXTURE with K = 2, K = 3, and K = 4.

The phylogenetic relationships among the Matou goat population and other participant goat populations are depicted in Figure 2. Each breed of NJ tree forms a distinct clade, showing variations in branch lengths that reflect genetic divergence. Particularly noteworthy is the close relationship observed between the Guizhou White goat and the Matou goat populations. This proximity is likely a result of their shared geographic origins in Guizhou and Hunan Provinces, facilitating genetic exchanges and fostering a closer kinship between these two breeds (Figure 2a). The PCA analysis revealed population stratification and showed the same trends as the NJ tree. Principal Component 1 (PC1) captures 2.31% of the genetic variation, while PC2 accounts for 1.98%. These findings indicate genetic differentiation between goats residing at high and low altitudes. Furthermore, the distribution patterns of Guizhou White goats and Matou goats appear to be dispersed, suggesting a potentially higher level of differentiation when compared to other goat breeds (Figure 2b). The admixture analysis results, as presented in Figure 2c, reveal distinct genetic clustering. By using ADMIXTRUE v1.3 to estimate the number of ancestral groups, the minimum value is obtained when the value of cv (cross-validation) is 3 (K = 3) (Table S4). It can be assumed that these seven groups mainly originated from three ancestral groups. Among them, the ancestral composition of the Matou goat is more complex, indicating a multi-ancestor population. However, across all ancestry component analyses, the Matou goat consistently stands apart from other goat breeds. It exhibits a closer genetic affinity with the Guizhou White goat, hinting at a multi-ancestral lineage within the Matou goat population.

### 3.3. Population Genetic Diversity

**Figure 3:**
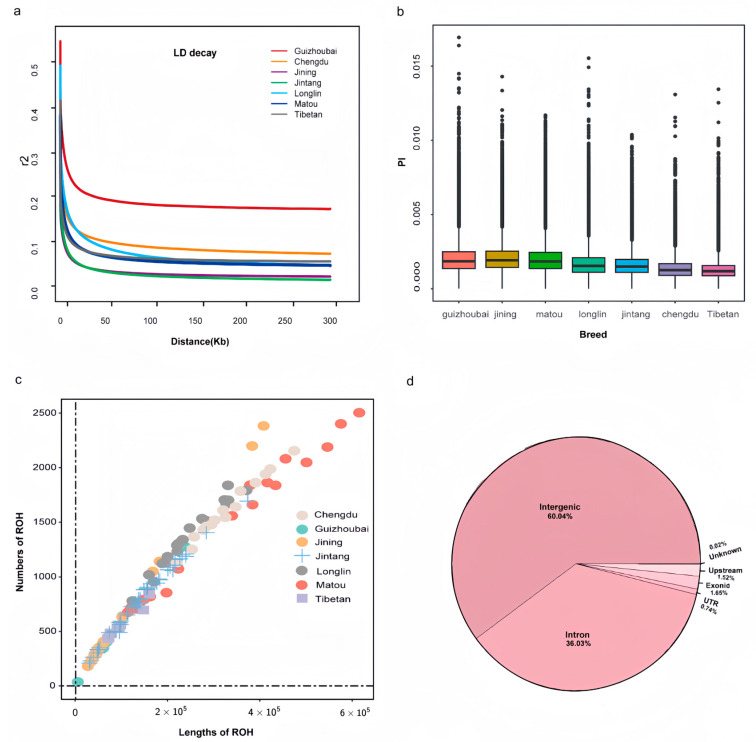
Genetic diversity among 153 samples from seven populations: (**a**) decay of linkage disequilibrium on goat autosomes estimated from each breed; (**b**) box plots of the nucleotide diversity for each group. The points that were on the outside of the whiskers showed outliers; (**c**) the estimation of the total number and length of runs of homozygosity (ROH) for each group; (**d**) functionally annotated distribution of polymorphic loci across all SNPs of Matou goat.

The results of the assessment of nucleotide diversity are presented in Figure 3a. Among the seven breeds analyzed, the Jining Grey goat exhibited the highest nucleotide diversity (0.00192). Additionally, the nucleotide diversity within the Matou goat population (0.00183) fell within a moderately lowered range in contrast to other studied goat populations. The results of the LD decay patterns can be visualized in Figure 3b, distinctions emerged among the different groups. Notably, Jintang Black and Jining goats exhibited the lowest LD levels, while Matou goats displayed intermediate LD levels, and Guizhou White goats demonstrated the highest LD levels, especially over greater inter-marker distances (>50 kb). Our analysis of runs of homozygosity (ROH) (Figure 3c) across all individuals revealed varied distributions of ROH within each group. In particular, Jintang and Tibetan goats generally displayed smaller ROH lengths and numbers compared to other groups. Conversely, the Matou goat group exhibited more extensive variability in ROH, with a predominance of longer and more numerous ROH segments.

### 3.4. Genome-Wide Selective Sweep Test

**Figure 4:**
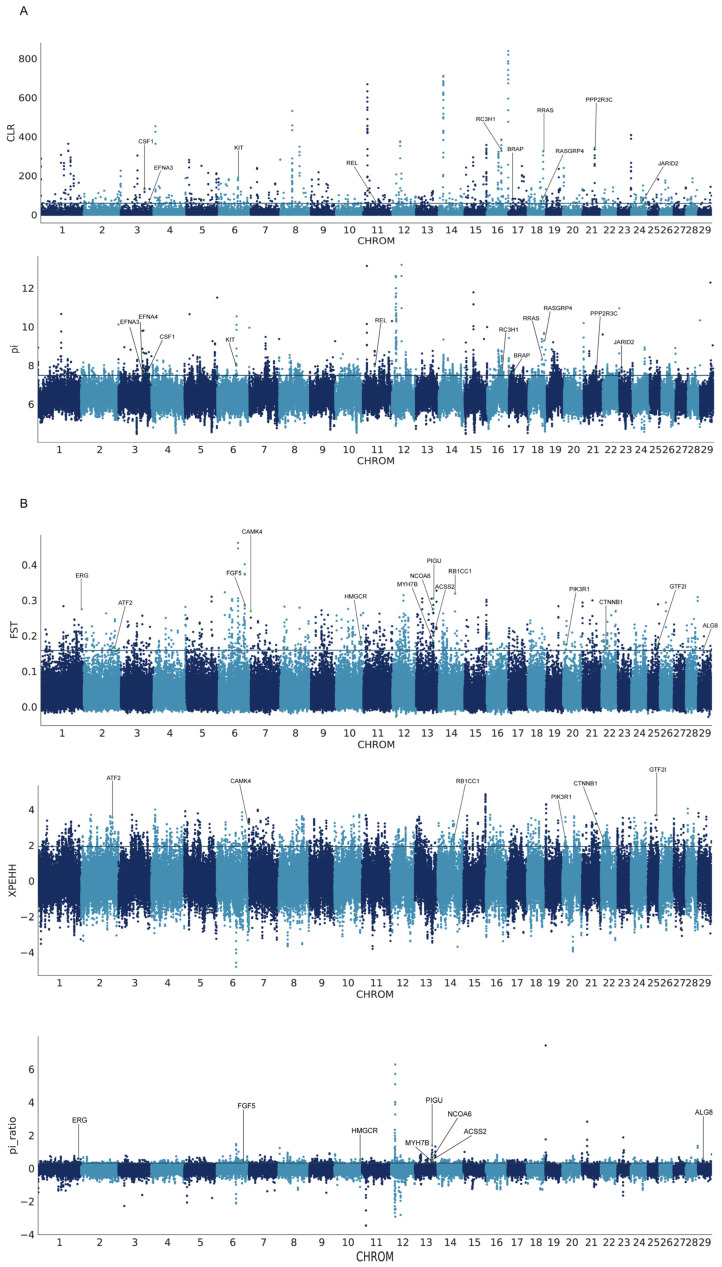
The signatures of positive selection in Matou goat: (**A**) Manhattan plot of selective sweeps by composite likelihood ratio (CLR) and θπ methods in Matou goat; (**B**) Manhattan plot of selective sweeps by Fst, π-ratio, and cross-population extended haplotype homozygosity (XP-EHH) methods between Matou goat and Jining Grey goat.

**Figure 5:**
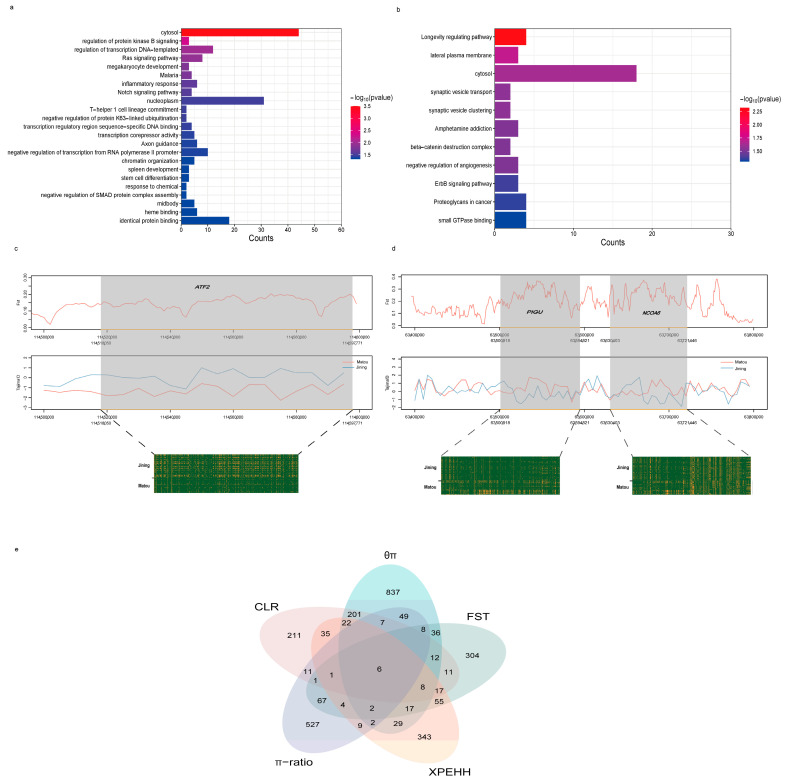
Candidate gene of positive selection between Matou goat and Jining Grey goat: (**a**) KEGG pathways and GO terms for these overlapped genes revealed by θπ and CLR; (**b**) KEGG pathways and GO terms for these overlapped genes revealed by Fst and XP-EHH; (**c**) Tajima’s D value and haplotype patterns heatmap of ATF2 gene region in Matou goat and Jining Grey goat; (**d**) Tajima’s D value and haplotype patterns heatmap of PIGU and NCOA6 genes region in Matou goat and Jining Grey goat; (**e**) number of candidate genes supported by five methods in each of the Venn diagram components between Matou and Jining Grey goat.

Our analysis, using θπ and CLR metrics, identified the top 1% of signals as candidate regions associated with genomic selection signatures in Matou goats(Figure 4A,B), resulting in the discovery of 1236 genes through θπ and 543 genes through CLR analysis (Tables S5 and S6). By intersecting these two methods, we obtained a set of 256 candidate genes (Figure 5e). Functional enrichment analysis using KEGG pathways and GO terms for these overlapped genes revealed significant enrichments, encompassing four KEGG pathway terms and 19 GO terms (corrected *p*-value < 0.05, Figure 5a and Table S7). Notably, the KEGG pathway-identified “Ras signaling pathway” featured prominently, including eight genes (*BRAP*, *EFNA3*, *RRAS*, *CSF1*, *KIT*, *REL*, *EFNA4*, *RASGRP4*). Additionally, among further elucidating positive selection characteristics between Matou goats and Jining Grey goats, we employed three methods (Fst, π-ratio, XP-EHH). Genomic regions identified by at least two methods (*p* < 0.005) were considered candidate regions of positive selection. The top 1% of signals were also selected as candidate regions. These analyses yielded 549, 694, and 1383 genes, respectively (Tables S8–S10). By intersecting the results of Fst and π-ratio, we identified 89 candidate genes, and by intersecting Fst and XP-EHH, we obtained a total of 100 candidate genes (Figure 5e). Subsequently, we conducted functional enrichment analysis for these 100 overlapping genes using KEGG pathways and GO terms (corrected *p*-value < 0.05, Figure 5b and Table S10). The “Longevity regulating pathway” emerged as the most significantly enriched pathway, featuring four genes (*ATF2*, *CAMK4*, *RB1CC1*, *PIK3R1*). Additionally, three genes (*ATF2*, *CTNNB1*, *GTF2I*) related to “negative regulation of angiogenesis” exhibited significant enrichment. Among the 89 candidate genes, although no significant pathways of particular interest were identified, functionally important genes were observed in categories such as adipose (*ACSS2*, *ALG8*, *MYH7B*, *PIGU*), hair color follicle (*NCOA6*, *FGF5*), skeletal (*ERG*), and cholesterol (*HMGCR*).

Notably, three genes—*ATF2*, *NCOA6*, and *PIGU*—stood out as especially significant, supported by Tajima’s D statistics and elucidated distinct haplotype patterning, indicating selection distinction between Matou goats and Jining Grey goats (Figure 5c,d). *ATF2* plays a pivotal role in lipid metabolism regulation [28], while *NCOA6* is linked to Matou goat coat color, and *PIGU* is involved in metabolic regulation [29], with potential functional similarities to *NCOA6* [30].

## 4. Discussion

The Matou goat is a local breed of the southern Chinese ethnic group and is regarded as the best goat breed for meat in China. However, there are few reports on its genetic diversity. Therefore, it is important to study the genetic resources of the Matou goat comprehensively. In this study, we investigated the genetic composition, relatedness, and selective signals related to adaptive traits of the Matou goat population using the genome sequence data of 20 Matou goats and 133 domestic goats. The results of NJ tree, PCA, and admixture analysis showed that Matou goats are distinguished from other goat breeds, that the ancestral composition of Matou goats is more complex, indicating a multi-ancestor population, and that they were closer to Guizhou White goats in terms of their genetic composition. We hypothesize that the unique geographic location of Hunan and Guizhou may have led to more artificial selection for the Matou goat, resulting in a complex genetic background. Population-based metrics (e.g., nucleotide diversity, LD, and ROH) within a breed reflect evolutionary factors such as natural/artificial selection, population history, and migration [31]. Matou goats had moderate levels of nucleotide diversity and LD. In ROH, Matou had high ROH values, which could indicate closer relatedness and higher inbreeding within the population, showing the impact of related matting patterns and breeding history of artificial selection.

In our study, we found that *BRAP*, *EFNA3*, *RRAS*, *CSF1*, *KIT*, *REL*, *EFNA4*, and *RASGRP4* are all located in the Ras signaling pathway by using GO and KEGG enrichment analysis in the genome of the Matou goat. The Ras signaling pathway widely exists in eukaryotic cells, and it is one of the important pathways that mediate cellular information transmission, which consists of three conserved signaling networks as follows: *MAPK*, *MAPK* kinase (*MKK*), and *MAPK* kinase (*MKKK*) [32,33]. This pathway mediates a variety of biological effects, including cell proliferation, differentiation, transformation, inflammation, and apoptosis. Specifically, the *BRAP* gene is essential for cardiomyocyte proliferation, and its expression controls cell cycle activity and prevents developmental arrest in developing cardiomyocytes [34]. *EFNA3* and *EFNA4* encode a member of the ephrin (*EPH*) family [35]. *RRAS*, an atypical member of the *Ras* subfamily of small GTPases, enhances integrin-mediated adhesion and signaling through a poorly understood mechanism. It regulates the organization of the actin cytoskeleton to sustain membrane protrusion through the activity of PL Cepsilon [36]. *CSF1* is a cytokine that controls the production, differentiation, and function of macrophages and promotes the release of pro-inflammatory chemokines and thus plays an important role in innate immunity and inflammatory processes [37,38,39]. Moreover, this gene also plays an important role in regulating osteoclast proliferation and differentiation, and in regulating bone resorption, which is necessary for normal bone development [40,41]. These aforementioned functional genes’ high signaling suggested their role in helping the Matou goat to produce good quality mutton and show efficient growth attributes and strong resistance. Coat color is an important trait in goats, and Matou goats are mostly white. The *KIT* gene encodes the mast cell growth factor receptor, which is important for melanocyte formation, maturation, proliferation, and migration [42,43,44]. It has been reported that the *KIT* locus is regulated by the dominant white coat color in pigs [45] and sheep [46]. However, in goats, it has also been reported that the expression amount and expression site of *c-KIT* are not the main factors contributing to the differences in coat color in black and white goats [47]. Nevertheless, *KIT* may be associated with white coat color in goats. *RasGRP4* is essential for mast cell development and is an activator of *Ras* proteins, which play an important role in inflammatory responses and immune activation [48].

Collectively, these genes located in the Ras signaling pathway may play important roles in the development of cardiomyocytes, nerves, blood vessels, epithelial tissue, immunity, bone formation, and coat color in the Matou goat. Additionally, the three genes (*PPP2R3C*, *RC3H1*, and *JARID2*) are involved in spleen development, suggesting that they may also play a role in the immunization process of the Matou goat through enrichment.

The Jining Grey Goat is the world’s finest breed of local goat for lambskin, mainly produced in Jining and Heze in Shandong Province [49]. The grey coat of the Grey goat is made up of two colors of hair, black and white, and appears grey in appearance. Both males and females have horns [49]. To understand the genetic differences between the two breeds, we identified some specific genes and pathways through GO and KEGG enrichment.

First, the significantly enriched “longevity signaling pathway” is a pathway that can be artificially interfered with in model organisms using mutants to significantly alter the aging process in animals [50]. Three pathways have been reported as follows: the insulin signaling pathway [51,52], the gonadal pathway [53], and the mitochondrial pathway [54]. These signaling pathways can not only affect the aging of animals, but are also involved in their daily metabolism, growth and development, and reproduction. For example, insulin and mitochondrial pathways are also associated with glucose metabolism and lipogenesis [52]. Moreover, the Wnt/β-linker protein signaling pathway is involved in the regulation of various functions such as cell proliferation, differentiation, survival, apoptosis, and cell migration [55,56,57,58]. *CTNNB1*, a core factor of the classic Wnt/β-catenin pathway, plays an important role in animal reproduction [59], insulin regulation [60], candidate genes regulating fiber growth characteristics, and cartilage differentiation. *PIK3R3* is a regulatory subunit of *PI1K*, and *PIK3R1*’s role in the regulation of insulin signaling plays an important function in metabolic homeostasis [61] and is involved in glucose and lipid metabolism [62]. *HMGCR* reductase is the rate-limiting enzyme for cholesterol synthesis and is regulated via a negative feedback mechanism mediated by sterols and non-sterol metabolites derived from mevalonate. *HMGCR* catalyzes the conversion of (3S)-hydroxy-3-methylglutaryl-CoA (HMG-CoA) to mevalonic acid, which has been defined as the rate-limiting step in the synthesis of cholesterol and other isoprenoids, thus playing a critical role in cellular cholesterol homeostasis [63]. In addition, we have identified genes associated with adipose muscle development. The *ACSS2* gene encodes a cytoplasmic enzyme that catalyzes acetate activation. *ACSS2* deficiency reduces dietary lipid absorption by the intestine and also perturbs the repartitioning and utilization of triglycerides from adipose tissue to the liver due to lowered expression of lipid transporters and fatty acid oxidation genes. In this manner, *ACSS2* promotes the systemic storage or metabolism of fat according to the fed or fasted state through the selective regulation of genes involved in lipid metabolism [64]. Likewise, major cardiac and skeletal muscle (*MYH*) genes have been studied for decades [65]. In this way, *MYH6* (*α-MyHC*) is known as the cardiac myosin isoform and is expressed in the mammalian heart. However, *MYH7* is also the predominant isoform expressed in slow skeletal muscle fibers (also known as type I fibers), and *MYH7* is present in certain specialized skeletal muscles [66]. Recent genomic analyses have identified *MYH7b*, which is expressed in a variety of specialized muscles in mammals [67]. In addition, *ATF2* is a member of the *ATF/CREB* family of transcription factors that is activated by stress-activated protein kinases such as p38. Extensive studies have shown that *ATF/CREB* proteins sense and respond to extracellular fluctuations in nutrient concentrations, hormone levels, and energy status, and act as transcription factors or cofactors that play a key role in the regulation of whole-body homeostasis in key metabolic tissues, especially the liver. For example, it is involved in the regulation of many aspects of lipid metabolic processes, in the regulation of various cellular processes of glucose metabolism, especially in the regulation of gluconeogenesis and insulin sensitivity, and, in addition to that, in the regulation of hepatocyte growth and proliferation in response to a variety of external stimuli [68]. In a previously reported work [69], the authors generated *dATF-2* knockdown Drosophila through the use of RNA interfering agents, which also further validated that *ATF2* is essential for the regulation of lipid metabolism.

Secondly, Matou goats are all polled, whereas all of the Jining Grey goats have horns, so it is significant to study the genes associated with horn status. In the three methods overlap, the *ERG* gene is common to the intersection set. A previous study [70] has demonstrated that *TMPRSS2*:*ERG* can directly regulate the expressions of three osteoblastic markers (*ALPL*, *COL1A1*, and *ET-1*) at the cell level [69], indicating that *ERG* is involved in bone metabolism. It has also been shown that polled goats are affected by heterozygous SVs (10.1 kb deletion and a 480 kb duplication) containing two genes, *KCNJ15* and *ERG*, and the study also illustrates that none of the SNPs detected in the region of interest are plausibly causal mutations [70]. This study is at the SNP level as well, thus it also explains the discrepancy problem of the detection of *ERG* with high Fst values when there is no obvious mutation in the haplotype heat map. But in any case, it can be demonstrated that the variation of *ERG* is closely related to the horn status.

Finally, *NCOA6* and *FGF5* are both associated with hair development in animals. *FGF5* is a member of the fibroblast growth factor family (*FGF*s), which is expressed in a variety of sites, including mammalian hair follicles, the nervous system, and the testis, as well as during embryonic development [71]. It has been found that the protein-encoding *FGF5* is secreted during the hair growth cycle and signals the inhibition of hair growth through a mechanism that blocks dermal cell activation [72]. Disruption of *FGF5* via *CRISPR/Cas9* in goats has been reported to cause hair fiber lengthening, leading to a 30% increase in cashmere production [73,74]. Notably, [75] also demonstrated the association of the *FGF5* gene with high-altitude acclimatization and long hair in various goat populations. Geographically, Jining Grey goats are in the north of China, while Hunan is in the south, and Matou goats live at relatively low elevations and have relatively high ambient temperatures. Matou goats are generally short-haired, whereas Jining goats are mostly long and thin-haired. It can be hypothesized that *FGF5* is also associated with thermal acclimatization in animals. Moreover, *NCOA6* is a transcriptional coactivator, and this protein is involved in hormone-dependent coactivation of several receptors, including prostaglandin-like, vitamin A-like, vitamin D3, thyroid hormone, and steroid receptors. *NCOA6* regulates hepatic gluconeogenesis by regulating glucagon/cAMP-dependent gluconeogenic gene transcription through interaction with *CREB* [76], and is also a candidate gene for regulating fiber color expression [29], which may be implicated in the formation of white coat color in Matou goats. *PIGU* is a component of the GPI transamitase complex. GPI modification, as an important form of protein modification, can secrete and locate non-transmembrane proteins to specific regions outside the plasma membrane cell, and participate in biological processes such as signal perception on the cell surface, cell adhesion, material transport, and metabolism [77]. *PIGU* is also closely linked to *NCOA6*, and has more pronounced SNP mutations presumably due to a similar function to the *NCOA6* gene [30] and related to metabolic regulation. Although the function of *PIGU* in goats is unclear, our study and previously published articles can inform future studies of the *PIGU* gene.

Altogether, we found candidate genes related to the meat quality and coat color of Matou goats, which also provides a scientific basis for explaining the excellent meat quality and the formation of white hair traits of Matou goats, but further verification is needed.

## 5. Conclusions

In conclusion, the comparative whole-genome sequence analysis of the Matou goat population revealed insight into its genetic intricacies. Our study elucidates that the Matou goat had moderate to lower genetic diversity and showed a resemblance to the Guizhou White breed phylogenetically. In addition, selective sweep analysis screened numerous genes related to cardiomyocytes, immunity, coat color, and meat quality. These results can provide a reference for further research in terms of functional validation of genes and future initiatives for future breeding practices, and we will continue to conduct further discussion on the relevant genes in future studies.

## Data Availability

Links to publicly archived datasets analyzed or generated during the study: PRJNA1046221—SRA—NCBI (https://www.nih.gov/).

## Supplementary Materials

The following supporting information can be downloaded: https://www.mdpi.com/article/10.3390/genes15070909/s1, Table S1: Summary of sequencing data; Table S2: List of additional goat samples for analysis of genetic background in BoHuai goat; Table S3: Distribution of SNPs identified in goat breeds; Table S4: Cross-validation (CV) errors for ADMIXTURE ancestry models with K ranging from 2 to 7; Table S5: A summary of genes from CLR in Matou goat; Table S6: A summary of genes from θπ in Matou goat; Table S7: KEGG pathway analysis and GO enrichment of Matou goat candidate genes overlapped by θπ and CLR methods after corrected; Table S8: A summary of genes from Fst in Matou goat; Table S9: A summary of genes from π-ratio in Matou goat; Table S10: A summary of genes from XP-EHH in Matou goat; Table S11: KEGG pathway analysis and GO enrichment of Matou goat candidate genes overlapped by Fst and XP-EHH methods after corrected.
